# Correction to: Patient on Peritoneal Dialysis Transfers to Hemodialysis: Causes and Associated Risks

**DOI:** 10.34067/KID.0000001276

**Published:** 2026-06-03

**Authors:** Nanti E. Adoukonou, Annabel Boyer, Thierry Lobbedez, Clémence Bechade, Antoine Lanot

In the study by Adoukonou *et al.*, “Patient on Peritoneal Dialysis Transfers to Hemodialysis: Causes and Associated Risks” (*Kidney360* 2025;6(4):583–594. doi:10.34067/KID.0000000732), corrections are needed.

The authors have noted an error within the legends for Figures 4–6. The figure legend should be listed as the following.

The legend for Figure 4 should be corrected from “Figure 4: Estimates from the Cox and Fine-Gray models for risk of transfer to hemodialysis due to psychosocial causes. Survival models adjusted on age, sex, diabetes, Charlson comorbidity index (CCI), and center size.” to “Figure 4: Estimates from the Cox and Fine–Gray models for risk of transfer to hemodialysis due to infections. Survival models adjusted on age, sex, diabetes, CCI, and center size.”

The legend for Figure 5 should be corrected from “Figure 5: Estimates from the Cox and Fine-Gray models for risk of transfer to hemodialysis due to infections. Survival models adjusted on age, sex, diabetes, CCI, and center size.” to “Figure 5: Estimates from the Cox and Fine-Gray models for risk of transfer to hemodialysis due to mechanical issues. Survival models adjusted on age, sex, diabetes, CCI, and center size.”

The legend for Figure 6 should be corrected from “Figure 6: Estimates from the Cox and Fine-Gray models for risk of transfer to hemodialysis due to mechanical issues. Survival models adjusted on age, sex, diabetes, CCI, and center size.” to “Figure 6: Estimates from the Cox and Fine-Gray models for risk of transfer to hemodialysis due to psychosocial challenges. Survival models adjusted on age, sex, diabetes, CCI, and center size.”

